# Tobacco-Amblyopia

**Published:** 1899-12-02

**Authors:** Clements Hailes

**Affiliations:** Assistant-Surgeon, Bristol Eye Hospital.


### HOSPITAL CLINICS AND MEDICAL PROGRESS.

### TOBACCO-AMBLYOPIA.

By CLEMENTS HAILES, M.D., F.R.C.S., C.M.Edin., Assistant-Surgeon, Bristol Eye Hospital.

THE principal toxin producing amblyopia is tobacco, or tobacco combined with excessive use of alcohol. The subjects of tobacco amblyopia are generally males over 30 years of age, who have been in the habit of smoking strong brands of tobacco, from half an ounce to one ounce even being the daily allowance.

The pernicious effects of this excessive smoking of tobacco seem to be increased by the use of alcohol, though undoubtedly teetotal smokers are also victims to the complaint. Insufficient sleep is an adjuvant, whether it arises from the nature of the patients' business only allowing them to get short periods of rest at a time, or from their habits causing them to sit up late smoking; in fact, burning the candle at both ends. Smoking on an empty stomach as in the early morning before breakfast or far into the night with nothing but alcoholic drink to stimulate, seems also to increase the tendency to this form of amblyopia.

Amblyopia is only a gradual effect of the excessive use of tobacco, and the manifestation of the complaint is often not preceded by an increase in quantity of the tobacco used, but gradually the individual becomes aware of the increasing dimness of his eyesight, of sensitiveness to light, so that he likes to sit in a shady room, to read by a shaded light, or to wear smoked glasses when he goes out. One eye is as much, or nearly as much, affected as the other, and the sight is easily fatigued; spectacles are purchased at the optician's, but the sufferer soon realises they are not much good to him, and that his inability to read, write, or use his near vision generally, is rapidly increasing; it is at this point that he generally consults his medical attendant. He may complain of an exhausted or asthenic condition, of dyspeptic symptoms, of palpitation of the heart, inability to work as hard as usual, but he will not forget to lay the greatest stress on his failure of sight.

The eyes look much the same as usual, the pupils slightly dilated. Testing the vision with Snellen's types shows a considerable departure from the normal; type that ought to be visible at 24 feet can only be seen at six feet distance; with one eye at a time the vision may be $\frac{6}{24}$ in one, and $\frac{6}{60}$ only in the other, and there is a corresponding failure in near vision; though he may be able to see small print momentarily, it will be with an effort that he cannot prolong. Glasses afford little if any improvement. Having found these symptoms in a heavy smoker, one should proceed to test his central colour vision. Bits of red and green wool do well enough for a preliminary test. If he cannot distinguish these colours we have obtained more confirmatory symptoms, and might next examine his fundus and the media of the globe with the ophthalmoscope. The appearance of the fundus in such a case is practically normal, though some hyperæmia of the optic disc may be noticeable. Having obtained this knowledge that there is nothing in the fundus visible to the eye, such as central choroiditis or a hæmorrhage, to account for the scotoma for red and green, we may, by carefully testing the fields of vision with the perimeter, discover that this scotoma is well defined, of an oval shape, and confined to the space between the optic disc and the macula lutea; that outside this area colours can be distinguished, and the perception of white objects is in all parts of the visual fields normal.

The prognosis of an attack of tobacco amblyopia is decidedly good; if the smoking habit be given up or considerably reduced, the alcohol drinking curtailed, and the patient put on suitable *régime* as regards diet and medication, he should rapidly improve, and in about three weeks should begin to realise that his vision is getting better. The necessary treatment must be continued for some time. There is a story told of a heavy smoker who would not follow the advice of his physician as to the total abstinence from tobacco necessary to effect a cure, and as he naturally did not get any improvement under this physician's care he was persuaded to consult another one; this latter, seeing how the land lay, said that he did not consider the total abstinence from tobacco a necessary adjunct to the cure, but he laid great stress on the kind of pipe to be smoked, and warned his recalcitrant patient that the only safe pipe for him was a brand-new churchwarden, and as this gentleman led an active outdoor life, in which he had been invariably accompanied by "my Lady Nicotine," the curtailment in the choice of pipe worked wonders with him, and after a few months, without realising that he had been deprived of his tobacco, he made a good recovery. The other day I came across a shoemaker, who said perhaps he put his pipe in his mouth fifty times a day, and could not give up his "bacca"; on inspecting his pipe I found it was of a pernicious type, and have yet to find out what the churchwarden will do for him, but he is not likely to want to fill and hold in his mouth a long churchwarden fifty times a day, while he is "clicking" his boots. The rest of the treatment consists in resting the eye as much as possible, that is to say, the retina, for the muscles of the eye are not affected. This is done by abstinence from close work and bright lights, by wearing smoked glasses, or a large shade. The excreta should be regulated, and an outdoor existence recommended. The patient should be prescribed strychnine, either as tincture of nux vomica, commencing with ♏iij. thrice daily, increasing the dose ♏iij. a day until physiological symptoms appear, and then keeping on with the dose suitable for some six or eight weeks. When the patient can attend regularly, a daily subcutaneous injection of the nitrate of strychnine, gr. 1-100th, with an interval every seventh day, is a more rapid way of treatment. It can also be given as the sulphate in pill form, 1-50th to 1-20th of a grain twice or thrice a day, or as the liquor strychninæ hydrochloridi ♏ij. to ♏v. three times a day; or in mild debilitated cases, with iron, quinine, and phosphorus as Easton's syrup.

Pathologically this form of amblyopia is due to a retrobulbar neuritis affecting a particular strand of nerve fibres which have their termination in the oval part of the retina marked out by the scotoma for red and green, situated between the macula lutea and the optic disc. On the cells from which these fibres are derived nicotine has a peculiar and particular influence. A similar condition has been attributed to bisulphide of carbon, also to stramonium when excessively smoked by asthmatics; but undoubtedly tobacco is the drug responsible for by far the larger majority of these cases of central toxic amblyopia.

To sum up, tobacco amblyopia occurs in men addicted to smoking strong tobacco in excessive quantities or under adverse circumstances, such as on an empty stomach or with a constitution lowered in vitality by the abuse of alcohol. The vision gradually dims; there is a loss of power to distinguish red and green-coloured materials when held close to the eye so that their form falls on the central visual field. No ophthalmoscopic changes are usually found, but the perimeter marks a well-defined scotoma, oval in shape, horizontal in position, extending from the outer side of the macula lutea to the blind spot, and of which the patient is not conscious; hence it is termed a "negative scotoma." Prognosis is favourable if the cause be removed, and medication with strychnine in one or other of its forms is the chief treatment.

